# Enhancing the Quality of Spray Application in IRS: Evaluation of the Micron Track Sprayer

**DOI:** 10.3390/insects13060523

**Published:** 2022-06-06

**Authors:** Janneke Snetselaar, Rosemary S. Lees, Geraldine M. Foster, Kyle J. Walker, Baltazari J. Manunda, David J. Malone, Franklin W. Mosha, Mark W. Rowland, Matthew J. Kirby

**Affiliations:** 1 Department of Disease Control, London School of Hygiene and Tropical Medicine, London WC1E 7HT, UK; mark.rowland@lshtm.ac.uk (M.W.R.); matthew_kirby@abtassoc.com (M.J.K.); 2 Innovative Vector Control Consortium, Liverpool School of Tropical Medicine, Liverpool L3 5QA, UK; david.malone@gatesfoundation.org; 3 Department of Vector Biology, Liverpool School of Tropical Medicine, Liverpool L3 5QA, UK; rosemary.lees@lstmed.ac.uk (R.S.L.); geraldine.foster@lstmed.ac.uk (G.M.F.); kyle.walker@lstmed.ac.uk (K.J.W.); 4 Pan African Malaria Vector Research Consortium, Kilimanjaro Christian Medical University College, Moshi P.O. Box 2240, Tanzania; manundabaltazari@gmail.com (B.J.M.); fwmosha@gmail.com (F.W.M.)

**Keywords:** IRS, application technology, broflanilide, clothianidin, deltamethrin, pirimiphos-methyl

## Abstract

**Simple Summary:**

A key tool in the fight against mosquitoes, which transmit malaria, is the application of insecticidal indoor residual spray (IRS) to the internal walls of buildings where mosquitoes alight and rest to digest their blood-meals. When evaluating the effectiveness of IRS formulations for killing mosquitoes when applied to a wall, it is important that the insecticide is applied evenly at the target dose. Traditionally, IRS is applied using a hand-held pump, but this study showed that an automated track sprayer delivered the desired dose to wall surfaces more accurately and more evenly. This was first shown using a fluorescent tracer to measure spray deposit on the wall of a laboratory, and then by spraying different IRS formulations onto the walls of an experimental hut.

**Abstract:**

Indoor residual spraying (IRS) has changed little since its introduction in the 1940s. Manual spraying is still prone to variation in insecticide dose. To improve the application of IRS in experimental hut trials, an automated track sprayer was developed, which regulates the speed of application and the distance of the nozzle from the wall, two key sources of variation. The automated track sprayer was compared to manual spraying, firstly using fluorescein solution in controlled indoor settings, and secondly in experimental huts in Tanzania using several IRS products. Manual spraying produced greater variation with both fluorescein and insecticide applications. Both manual and automated spray methods under-dosed the actual dose sprayed compared to the target dose. Overall, the track sprayer treats surfaces more consistently, offering a potential improvement over manual spraying for experimental hut evaluation of new IRS formulations.

## 1. Introduction

Indoor residual spraying (IRS) is a cornerstone of malaria vector control. It is typically conducted manually by spray operators using compression sprayers, a method that has seen little change since its introduction in the 1940s. Equipment specifications for IRS were first published in 1964 and described a hand-operated cylindrical tank with a hose, lance, and a flat-fan nozzle [1]. Specifications remained relatively unchanged until the addition of a control flow valve (CFV). Regulating the flow of insecticide through the nozzle with a CFV meant that, despite decreasing pressure in the tank while spraying, the emitted spray volume stayed constant. The introduction of CFVs resulted in a recommended application rate of 30 mL/m^2^, rather than the original 40 mL/m^2^, reducing the volume (and thus weight) of water needed to spray a surface area. A second change was the shift from stainless steel nozzles to a more durable ceramic nozzle, reducing the risk of inconsistent spray due to wear on the nozzle [2].

Although these innovations in spray equipment have resulted in more accurate IRS applications, the spray technique itself has remained similar to that in early spray campaigns [3]. In control campaigns, manual spraying is prone to variation in the dose applied due to variation in competence and skills, and lapses in concentration between operators. Even in experimental hut trials, human error by individual spray operators can result in large differences in insecticide application rates between walls within a house and between positions on a single wall. Both overdosing and underdosing of IRS products have been reported during the conduct of experimental hut [4,5,6,7], highlighting the challenges in spraying IRS products accurately and consistently. Overdosing of IRS products can lead to higher than anticipated costs and potential safety concerns, while underdosing can result in a shorter residual half-life and development of mosquito resistance due to exposure to sublethal doses [8,9]. Consistency in the speed of application and the distance of the sprayer nozzle from the surface are critical to applying the correct dose of insecticide to walls, ceilings, and other sprayable surfaces.

High-quality training and supervision of spray operators, plus good maintenance and calibration of spray equipment, can contribute to the accurate application of IRS products in experimental hut studies (and in IRS spray campaigns). Accurate quality control of spray application, however, remains challenging and relies on complicated, timely, and expensive technology such as HPLC analysis of sprayed filter papers, or methods that are relatively insensitive to variations in dose such as cone assays. Variation in insecticide delivery has impacted the effectiveness of spray campaigns or outcome of regulatory trials. High accuracy in the measuring of spraying is particularly important in experimental hut trials evaluating different dosages of IRS products [10,11,12,13,14,15], as these trials need to inform development decisions on the most appropriate application rate for novel products prior to regulatory evaluations and subsequent market launch. To improve the consistency of the application of IRS products, the automated mechanical track sprayer was developed by Micron Sprayers Ltd., with support and funding via the Innovative Vector Control Consortium (IVCC). The track sprayer was designed specifically for experimental use, aiming to improve the quality of insecticide application in semi-field experimental hut studies.

This comparative study was conducted to evaluate whether the application of IRS products by mechanical track sprayer gives less variation in spray application rate than conventional manual spraying. The comparison was made during two experimental phases: the first phase, conducted in the laboratory at the Micron Centre in Herefordshire, England, used fluorescein diluted in water; and the second phase, under semi-field conditions at the Kilimanjaro Christian Medical University College (KCMUCo, Moshi, northern Tanzania) used IRS products. The proof-of-concept laboratory phase allowed for a high-throughput and low-cost comparison of both spray methods, whereas the semi-field phase provided the opportunity to test with insecticidal products under more realistic experimental conditions. For both phases, the manual spraying was carried out by an experienced spray operator and the mechanical spraying was conducted using the automated track sprayer.

## 2. Materials and Methods

### 2.1. Spray Methodology

**Figure 1:**
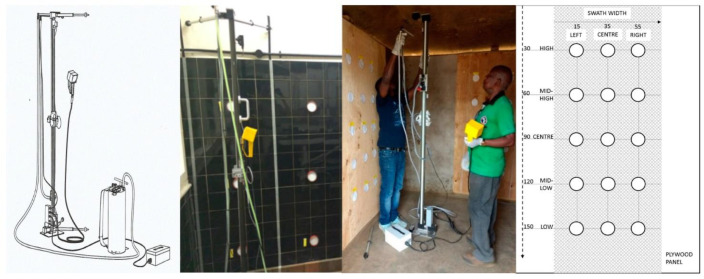
A schematic overview of the sprayer adapted from the Micron product manual is shown on the (**left**). A photo showing the set-up of track sprayer in the lab (**centre left**) and in an experimental hut (**centre right**) is shown, and a schematic overview of the filter paper positions on plywood panels, with values in centimetres, is shown on the (**right**).

The Micron Track Sprayer (Micron Sprayers Ltd., Bromyard, UK) consists of a conveyer belt along which a spray head with nozzle can move vertically up and down (Figure 1). The speed of the nozzle is adjustable using an electronic hand-held controller, and power is provided by a rechargeable battery pack. The spray head is connected to a pressurized spray tank of the Micron compression sprayer. Extendable arms at the top and bottom of the track sprayer were set 45 cm from the wall surface, and the equipment was levelled horizontally using a standard spirit level. The travelling speed was set to 0.45 m/s, which corresponds to 1 m sprayed every 2.2 s as per WHO guidelines [2].

The Micron track sprayer was compared to manual spraying, performed as detailed in WHO guidelines [2,16]. Before manual spraying, the spray operators were extensively trained using standard operating procedures (SOPs) based on the WHO guidelines which details lance speed and angle, distance from the wall, and speed of movement vertically up and down the walls during application. Spray tanks were calibrated and maintained according to good laboratory practice (GLP) standards. Sprayers were calibrated and deemed acceptable when spraying 550 mL ± 10% per minute. Spraying was carried out at an application rate of 30 mL/m^2^ using a 1.5 bar CFV. To ensure comparability between the two spraying application methods, the same spray tank, insecticide solution, 8002E flat fan nozzle, and CFV were used for both manual spraying and automated track spraying.

For both phases of the comparison, a similar protocol was used; minor differences between the round with fluorescein and the round with insecticides are detailed below.

### 2.2. Spraying of Fluorescein

The first phase of spraying was performed using 0.1% *w*/*v* fluorescein sodium salt diluted in water. A 3.55 m × 2.00 m tiled wall surface was marked up to accommodate five 75 cm spray swaths (70 cm spray + 5 cm overlap). Each set of five swaths constituted one replicate test. Filter papers (10 cm diameter, Whatman No.5) were held in place in Petri dish lids using a plastic ring; the lids were attached to the wall surface using self-adhesive Velcro strips in a grid pattern with three horizontal positions and five vertical positions per swath (see Figure 1). Vertical positions were located at the following heights: 1.80 m (high), 1.40 m (mid-high), 1.00 m (centre), 0.60 m (mid-low), 0.20 m (low). Horizontal positions were at 0.2 m (left), 0.375 m (centre), and 0.55 m (right) from the left edge of the swath. Six replicates using the track sprayer and five replicates spraying manually were performed using the fluorescein water solution. In total, 1045 filter papers were analysed. Track spraying was performed using a Micron CS10 compression sprayer tank. The target spray rate was 2 metres in five seconds; a metronome app was used to assist the manual spray person to follow an even spray rhythm. The spray time and direction (upwards or downwards) was recorded for each swath.

### 2.3. Spraying IRS Products

Three IRS products containing different active ingredients were sprayed in experimental huts: broflanilide (VECTRON^TM^ T500, Mitsui Chemical Agro Inc., Tokyo, Japan, batch no 18I-3671), pirimiphos-methyl (Actellic^®^ 300CS, Syngenta, Basel, Switzerland, batch no BSN9A2383), and a deltamethrin + clothianidin combination product (Fludora Fusion^®^, Bayer AG, Leverkusen, Germany, batch no EQ13001804). Target application rates were 100 mg/m^2^ for broflanilide (BRF), 1000 mg/m^2^ for pirimiphos-methyl (PMM), 200 mg/m^2^ for clothianidin (CTD), and 25 mg/m^2^ for deltamethrin (DLT). A different spray tank (Micron CS14) was used for each insecticide product. For both application methods, the spray tanks were positioned stationary on the floor, which differs from the WHO guidelines for manual spraying where the tank is typically carried over one shoulder. For each insecticide, four panels were sprayed with the track sprayer and four panels were sprayed manually (Figure 1). Filter papers (9 cm diameter Whatman No. 1) were fixed inside Petri dish lids with sticky tack; the lids were pierced in the centre and attached to the panel using shoe tacks. Filter papers were positioned in a grid as shown in Figure 1, with three horizontal and five vertical positions per swath. Each panel with 15 filter papers constituted one replicate test, resulting in four replicates per insecticide product.

### 2.4. Determining Spray Deposit Using a Fluorescent Tracer

Filter papers sprayed with a fluorescent tracer were removed from Petri dishes using tweezers and placed, with minimal handling, into individual labelled ziplock bags. 100 mL of 10% NaOH *v*/*v* solution was added to each bag and subsequently stored in the dark for 60 min. Each bag was agitated thoroughly for approximately 1 min to mix the solution and ensure all fluorescein had been extracted from the filter papers. Then, an aliquot of the sample was added to a glass test tube. Fluorescence of each sample was measured using a Sequoia–Turner Model 450 Fluorometer and fluorescein filter set with excitation at 490 nm and emission at 515 nm. The fluorimeter was calibrated before each replicate against known concentrations of fluorescein applied to filter papers. Before analysing samples, a single concentration standard was used to check for any drift in the fluorescence measured over time.

Fluorescence heat maps were generated using Microsoft Excel as a proxy for dosage applied. A three-colour format was used, with the lowest recorded fluorescence value as the minimum (yellow), the second highest recorded fluorescence value as the maximum (red), and a mid-point at 50% of the difference between the high and low points (blue) when recording concentration.

### 2.5. Insecticide Sprayed Filter Papers

Sprayed filter papers were left to dry in the experimental huts for a minimum of 24 h, before they were wrapped individually in aluminium foil and stored at 5 ± 3 °C. The concentration of active ingredient on the filter papers was determined using high-performance liquid chromatography (HPLC). Samples were extracted from the filter papers at KCMUCo, and dried extracts were shipped to the Liverpool School of Tropical Medicine (LSTM) for HPLC analysis. The HPLC analysis was performed on a Dionex UltiMate 3000 comprising of an autosampler, quaternary pump, and variable wavelength detector. Chromeleon 7.2 SR4 software was used for peak analysis.

Prior to extraction, 12 circles were punched out of the filter paper using a 0.635 cm radius (½ inch diameter) hole punch, to have a consistent exact surface area of 15.201 cm^2^ per disc to extract the sample from. A volume of 5 mL of a 100 µg/mL DCP in acetone solution was pipetted into a glass tube containing each filter paper sample and sonicated for 15 min using an Ultrawave U500H Ultrasonic Cleaning Bath (4.5 litre). Then, 1 mL of the sonicated sample was transferred to a new vial and left to evaporate until dry.

Samples were re-suspended using 1 mL of HPLC grade acetonitrile, and vortexed for at least 1 min at 2500–3000 rpm. Subsequently, samples were centrifuged (Eppendorf Centrifuge 5430) at 13,000 rpm for 20 min, and directly afterwards 100 µL of each sample was pipetted into individual HPLC vials. A 250 mm × 4.6 mm HPLC column (Thermo Scientific Hypersil Gold C18) was used for all active ingredients, using an injection volume of 20 µL. HPLC methods were tailored for each active ingredient as detailed in Table 1.

### 2.6. Statistical Analysis

Graphical output was generated using R version 4.0.5 using the ggplot2 package and Microsoft Excel. Spray data for both track and manual spraying were not normally distributed, even after transformation with either log_10_ or square root methods. Therefore, untransformed data were used with non-parametric tests for statistical analysis. Significance between spray categories was evaluated using the Kruskal–Wallis method, incorporating Dunn’s correction for multiple comparisons, with an alpha of 0.05. Unpaired, 2-tailed *t*-test was used to compare time taken to spray a downwards swath vs. an upwards swath.

A fluorimetry calibration was performed with each trial, and data were corrected accordingly prior to analysis. The fluorescein spray data were analysed with and without overlap points, due to the assumed greater inherent variability in the overlap spray zones. Comparisons between the insecticide concentration and the target dose, and subsequently the corrected target dose, were performed using one-sample Wilcoxon signed rank tests. Analysis was performed using Microsoft Excel, R version 4.0.5, and GraphPad Prism v7.03.

## 3. Results

### 3.1. Fluorescein Spray Results

In total, 547 and 488 filter papers sprayed with the automated track sprayer were included for analysis with and without overlap points, respectively, and 475 and 375 filter papers were included for the manual spray.

**Figure 2:**
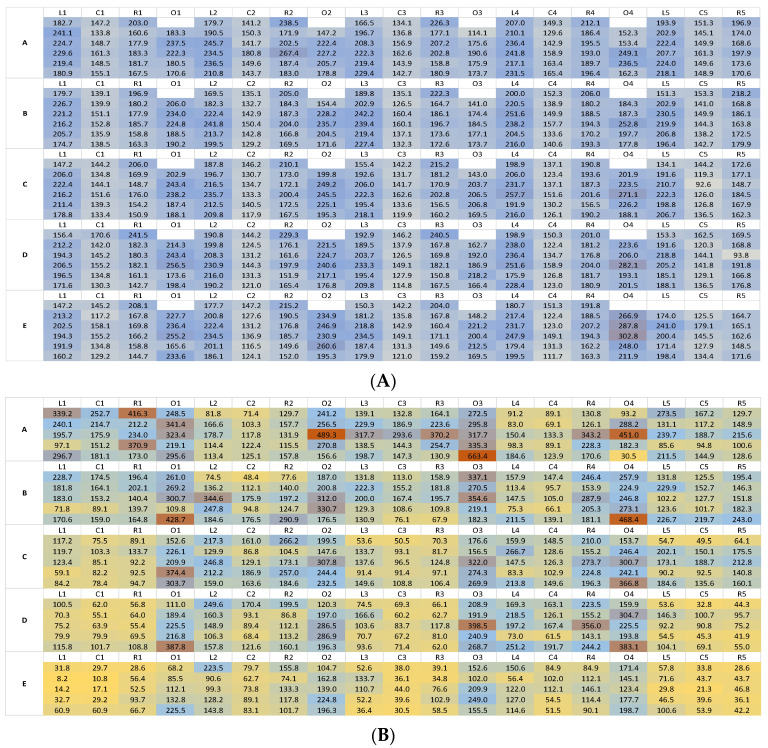
Heat maps of fluorescein deposition on wall surfaces using an automated track (**A**) and manual (**B**) spray. Filter papers to collect fluorescein deposited by each spray type were attached to walls in the configuration shown. Five swaths were present on each wall, measured at the Left (L), Centre (C), Right (R), and Overlap (O) positions. Letters A–E indicate the height position down the wall. Six trials were performed for the track spray and five were performed for the manual spray. Individual cells show the corrected fluorescence values for each spray trial replicate. Orange indicates higher fluorescein values, blue indicates midpoint values, and yellow indicates low values. A far higher degree of variation in fluorescein deposition is apparent in the manual spray compared to the track spray.

Heat maps plotting the distribution of fluorescein deposition on the wall surface showed a general uniformity of spray from the automated track sprayer, with the majority of recorded values falling around the mid-point colour range; some visual variation was apparent between the Left, Centre, and Right swath positions (Figure 2A). Similar uniformity of deposition was not evident in the manual wall spray, which showed high variability of spray over the entire wall surface, particularly when fluorescein deposition at the top and bottom wall positions were compared (Figure 2B).

Descriptive statistical analyses confirmed that wall spraying using the track sprayer had higher median and mean fluorescein deposition, and lower standard deviation and percentage coefficient of variations in the track spray compared to the manual spray (Table 2, SD of 36.15 and 33.87 for track spray with and without overlaps, respectively, compared to 88.83 and 69.51 for manual spray with and without overlap, respectively, and coefficient of variation of 19.96% and 19.30% for track spray with and without overlap, respectively, vs. 58.03% and 53.54% for manual spray with and without overlap, respectively).

Including the overlap positions resulted in a significant different dataset for manual spraying (*p* ≤ 0.0001), but not for the track sprayer dataset (*p* = 0.6998). Both sets of manual spray data were significantly different to the track spray datasets (4 comparisons, *p* ≤ 0.0001 in all cases). Analysis of each combination of vertical and horizontal swath positions showed greater variation of fluorescein deposition in the manual spray at every wall position point compared to the track sprayer.

### 3.2. Insecticide Spray Results

**Figure 3:**
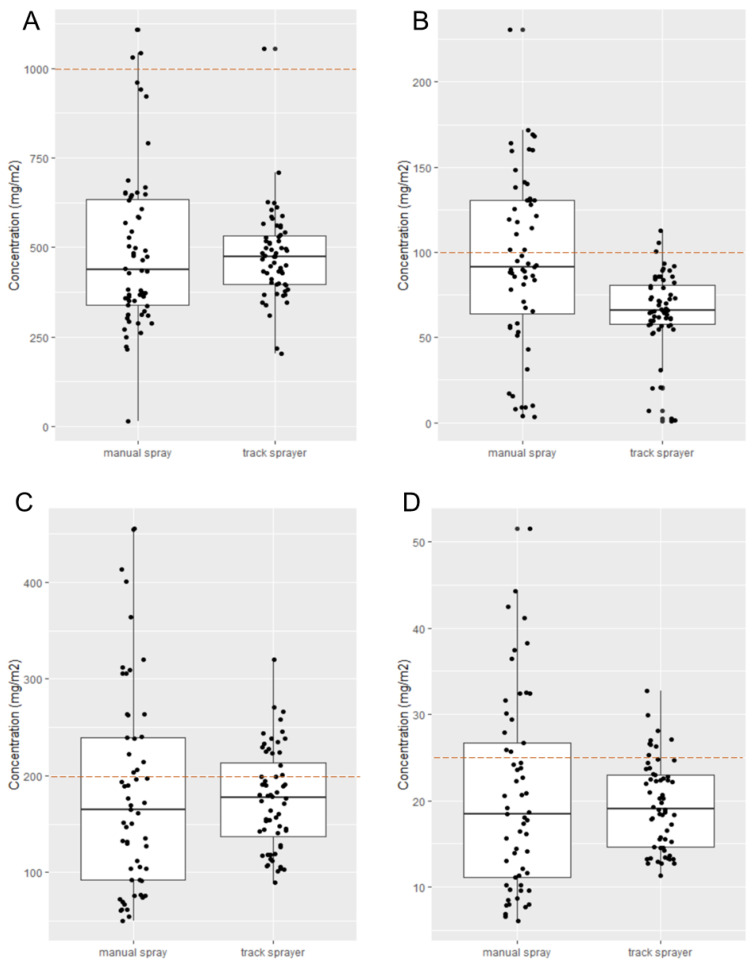
Concentration of active ingredient on filter papers sprayed by manual spraying or the automated track sprayer. Filter papers were attached to plywood panels in a grid of 15 per swath. Four swaths were treated for each spray method per insecticide, resulting in 120 filter papers per insecticide. Letters A–D indicate the active ingredients; pirimiphos-methyl (**A**), broflanilide (**B**), clothianidin (**C**), and deltamethrin (**D**). Individual dots show the values for each filter paper. Boxplots indicate median, 25th and 75th percentile, extreme lines, and potential outliers. Dashed horizontal lines represent the target concentration for each insecticide.

For both pirimiphos-methyl (PMM) and broflanilide (BRF), 60 filter papers were sprayed with the track sprayer and 60 with manual spraying. As a dual AI formulation, the concentration of both deltamethrin (DLT) and clothianidin (CTD) was determined in the same filter papers; 57 for manual spraying and 60 for the track sprayer. The concentration of active ingredient on each filter paper was visualised in boxplots (Figure 3). Similar to spraying with fluorescein in laboratory conditions, variation in insecticide application rate in the experimental huts was much greater for manual spraying compared to the track sprayer. For both PMM and BRF, Levene’s test reported unequal variances between manual spraying and the track sprayer (F = 3.316, *p* < 0.001 and F = 4.3533, *p* < 0.001, respectively), indicating that the variation in insecticide application rate from top to bottom and from left to right of the wall was larger when spraying manually compared to using the automated track sprayer. Likewise, for CTD and DLT, the vertical variance between the two application methods was statistically different (F = 4.1735, *p* < 0.001 and F = 4.6389, *p* < 0.001 respectively).

Two of the active ingredients, PMM (Figure 3A) and BRF (Figure 3B), showed a significant difference (*p* < 0.01 and *p* < 0.0001, respectively) in the sprayed concentration when comparing track and manual spraying. For BRF, this resulted in a lower median concentration for the track sprayer (66.0 mg/m^2^, SD 23.8) compared to manual spraying (91.4 mg/m^2^, SD 49.7), whereas for PMM the median track sprayer concentration was higher (485.8 mg/m^2^, SD 193.4) compared to manual spraying (392.3 mg/m^2^, SD 268.6). Clothianidin (CTD) and deltamethrin (DLT) were sprayed together in the combination product Fludora Fusion and, unsurprisingly, the results for the two actives followed the same pattern and trend (Figure 3C,D). The amount of CTD sprayed by manual application (177.9 mg/m^2^, SD 51.3) was not significantly different from the amount of CTD sprayed using the track sprayer (165.4 mg/m^2^, SD 104.7), *p* = 0.5288. Likewise, the amount of DLT sprayed by manual application (18.4, SD 11.0) was not significantly different from the amount of DLT sprayed by using the track sprayer (19.1, SD 5.1), *p* = 0.6217.

For each active ingredient, the amount delivered by each spray method was also compared to the target dose. For PMM the target dose is 1000 mg/m^2^, and both spray methods resulted in a significantly lower dose on filter papers (*p* < 0.0001). The concentration found in the liquid samples taken from the spray tanks prior to spraying was used to correct for possible mixing errors (see Table 3). This resulted in calculated concentrations of 568.5 mg/m^2^ for manual spraying and 576.6 mg/m^2^ for the track sprayer. Compared to the corrected target dose, manual spraying was significantly lower (*p* < 0.001), but there was no significant difference for the track sprayer (*p* = 0.07913).

For BRF, the amount of active ingredient sprayed on filter papers was not significantly different from the target dose for manual spraying (*p* = 0.4034, and *p* = 0.2403 for the corrected dose), but was significantly lower for the track sprayer (*p* < 0.0001). Similarly, for CTD, the dose applied to filter papers by manual spraying was not significantly different from the target dose (*p* = 0.0676, and *p* = 0.8863 for the corrected dose), but the dose applied by the track sprayer was significantly lower (*p* < 0.001, and *p* < 0.01 for the corrected dose). For DLT manual spraying was significantly different from the target dose of 25 mg/m^2^ (*p* < 0.01) but not after correcting for the concentration in the spray tank (*p* = 0.9051). The track sprayer resulted in a significantly lower dose, both corrected and uncorrected (*p* < 0.0001).

### 3.3. HPLC Analysis of Liquid Samples

Samples of insecticide solutions were taken directly from the spray tank before and after spray application for both application methods to be able to detect non-homogeneous mixing. Deviation from the target concentration was calculated for each active ingredient (Table 3). The target concentrations for the liquid samples were calculated by taking the recommended dose per m^2^, divided by the application rate of 30 mL/m^2^, resulting in a target concentration of 33.33 mg/mL for PMM, 6.66 mg/mL for CLT, 0.83 mg/mL for DLT, and 3.33 mg/mL for BRF. Apart from the concentration of BRF before spraying, the concentration of active ingredient found in the spray solution was generally lower than the target concentration. The spray tank solution had a DLT concentration of 0.65 mg/mL before and 0.71 mg/mL after manual spraying (target 0.83), and a CTD concentration of 5.66 mg/mL before and 6.15 mg/mL after spraying (target 6.66 mg/mL). The average concentration of BRF was 3.44 mg/mL before and 3.06 mg/mL after spraying, both within 10% of the target concentration (3.33 mg/mL). The concentration of PMM in the tank solution was considerably lower than the target dose (33.3), ranging between 19.22 mg/mL (−36%) and 15.11 mg/mL (−50%).

**Table 3:** HPLC results of liquid samples taken from the spray tank before and after spraying. The target dose in mg/m^2^ is given for each active ingredient. HPLC results are represented as a percentage deviation from the target dose.

		Concentration before	Concentration after	Target Concentration	Deviation from Target Dose
**PMM**	Manual	18.95 mg/mL	15.11 mg/mL	33.33 mg/mL	−37% to −50%
	Track	19.22 mg/mL	16.18 mg/mL	33.33 mg/mL	−36% to −46%
**CLT**	Manual	5.66 mg/mL	6.15 mg/mL	6.66 mg/mL	−8% to −15%
	Track	6.59 mg/mL	6.60 mg/mL	6.66 mg/mL	1%
**DLT**	Manual	0.65 mg/mL	0.71 mg/mL	0.83 mg/mL	−14 to −22%
	Track	0.79 mg/mL	0.77 mg/mL	0.83 mg/mL	−5% to −7%
**BRF**	Combined	3.44 mg/mL	3.06 mg/mL	3.33 mg/mL	3% to 8%

### 3.4. Consistency of Wall Spraying across the Swath

**Figure 4:**
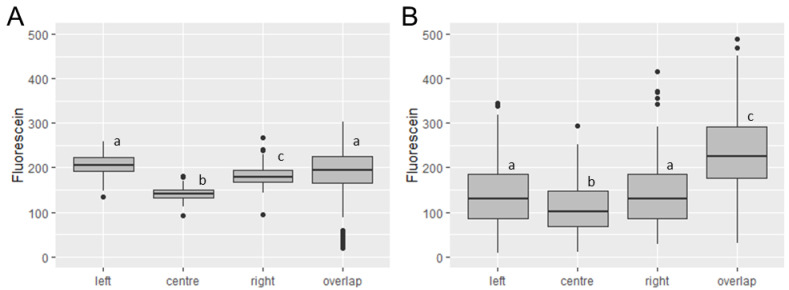
Box and whisker plots of horizontally stratified fluorescence. Track (**A**) and Manual (**B**) spray data was classified using left, centre, right, and overlap horizontal swath positions. The centre position on the track sprayer showed the most consistent fluorescein deposition. Greater differences were seen between horizontal positions on the track sprayer than the manual spray. Significant differences are indicated by different lowercase letters.

The nozzle delivered the most consistent fluorescein deposition from the track sprayer at the centre of the swath, displaying the lowest range of fluorescein deposited, the lowest standard deviation (13.43 vs. 24.25, 22.36 and 35.36 for Centre, Left, Right, and Overlap positions, respectively), and the lowest coefficient of variation (9.58% vs. 11.79%, 12.37% and 17.15% for Centre, Left, Right, and Overlap positions, respectively). However, significantly less fluorescein was deposited in this position compared to both the left and the right positions on the swath (*p* ≤ 0.0001 in both cases, Figure 4A). Further significant differences were seen between the Left and Right, Right and Overlap, and Centre and Overlap positions (*p* ≤ 0.0001 for all). No significant difference was seen between the Left and the Overlap positions (*p* ≥ 0.9999).

In contrast, there was no significant difference seen between the left and right swath positions when spraying manually (*p* ≥ 0.9999, Figure 4B). However, significant differences were seen between Left vs. Centre (*p* = 0.0041), Right vs. Centre (*p* = 0.0027), Left vs. Overlap (*p* = 0.0001), and Right vs. Overlap (*p* ≤ 0.0001) positions on the swath.

To discern whether the greater variation between fluorescein deposition at different wall heights in the manual spray was obscuring horizontal differences, data were further split into vertical and horizontal spray position categories. Except for comparisons including Overlap data, only one comparison showed a significant difference (CB vs. RB, *p* = 0.048). Plotting the stratified data showed a general trend for mean values from the Centre position to be lower than either Left or Right position data, indicating that this variation exists independently of the method of spray application.

Similar to results with fluorescein, the amount of insecticide sprayed with the track sprayer was most consistent in the middle position of a swath (Table 4). For both CLT and DLT, the middle position also had the lowest concentration deposited (*p* < 0.0001 for all combinations). However, this was not observed for PPM or BRF. For three out of the four insecticides, the left position on a swath resulted in a higher concentration of active ingredient applied compared to either the centre or right positions.

Greater variation in spraying was observed when spraying manually for all horizontal positions for all active ingredients, except for PMM in the left position. For PMM and BRF, there was a trend from left to right with a lower amount deposited at the left compared to the right position on a swath (*p* < 0.05 for PMM and *p* < 0.0001 for BRF). No significant differences were found for DLT. For CLT, the left position showed a significantly higher concentration of active ingredient applied compared to the middle position on a swath (*p* < 0.05).

### 3.5. Consistency of Wall Spraying along the Swath

**Figure 5:**
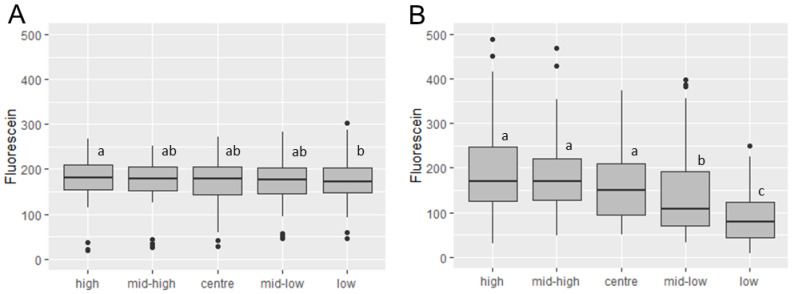
Wall height analysis for track and manual spray with fluorescein. (**A**) Track spray stratified by height with no overlap; (**B**) Manual spray stratified by height with no overlap. Significant differences are indicated by different lowercase letters.

Although the difference was slight, there was a general trend for fluorescein deposition from the track sprayer to be greater at the top of walls, decreasing with each vertical wall position (Figure 5). There were no significant differences between deposition onto filter papers immediately above or below each other. However, a significant difference between deposition onto filter papers at the uppermost and lowermost wall positions was evident (high vs. low, *p* = 0.0166).

Fluorescein deposition using a manual spraying (Figure 3B) showed significant differences between the two upper positions and the two lowest positions on the wall (four comparisons, *p* ≤ 0.0001). Significant differences in fluorescence were seen between the centre and the lower middle position (*p* = 0.0294), centre and bottom position (*p* ≤ 0.0001), and lower middle and bottom position on the wall (*p* = 0.0017). No significant differences were seen between the three upper positions. When the direction of spray (upwards or downwards swath) and total swath spray time were added to the analysis, the pattern of variation was preserved only in downwards swaths. Upwards swaths showed no significant difference between fluorescein deposition at the tops and bottoms of walls (*p*= 0.9555), but significant differences were seen between the top and centre of the wall (*p* = 0.0164) and the upper middle, middle, and lower middle compared to the bottom of the wall (*p* = 0.0014; *p* ≤ 0.0001; *p* = 0.0356, respectively). No significant difference was found between the time taken to spray a downwards swath vs. an upwards swath (*p* = 0.8779), indicating that it is spray rhythm, rather than spray time, that differs with spray direction.

The minor trend towards decreasing fluorescence with vertical positions was not shown when spraying with insecticides. No significant differences were found between vertical positions sprayed with the track sprayer for either PMM or BRF. For DLT and CLT, only the upper middle position was significantly different, compared to the lower middle position (CLT; *p* < 0.05) or the centre position (DLT; *p* < 0.05).

Variation in insecticide application rate when sprayed manually was generally larger by vertical position compared to the track sprayer. Although some significant differences were found, there was no common trend between insecticides (Table 5). For PMM, the centre position had a significantly lower concentration on filter papers compared to the upper middle and lowest position (two comparisons; *p* < 0.05). No significant differences were found for BRF. For DLT, only the highest position was significantly different, compared to upper middle, centre, and lower middle positions (three comparisons, *p* < 0.01) and lowest position (*p* < 0.001). Similarly, for CLT the highest position was significantly different, compared to upper middle and centre positions (two comparisons; *p* < 0.01) lower middle position (*p* < 0.05), and lowest position (*p* < 0.001).

## 4. Discussion

IRS is a widely applied vector control intervention. However, relatively little attention is given to the assessment of application rates whether at the level of control programmes and communities, or at the level of households. Tools aimed at improving consistency of application on a community level, such as the IK Smart Light [17], are being developed but are not yet widely deployed. Apart from the implications for IRS campaign success, consistent delivery of insecticidal products is important when conducting experimental hut trials, which are reliant on the well-defined application rate of IRS products. In this study, we compared an automated track spray system for IRS to a well-trained human spray operator, to discern whether the track sprayer delivered a more consistent spray such that use in experimental hut trials could be implemented.

Large variation in spray deposits, measured by the amount of insecticide applied to a filter paper, have previously been reported for the three insecticides tested in this study, ranging from 0.31 to 3.78 times the recommended dose for Actellic 300CS [18], between 0.63 and 1.37 times the dose for Vectron T500 [12,13], and between 0.80 and 1.32 for Fludora Fusion [19,20]. A large proportion of this variation could be removed by using an automated spraying process, such as shown here with the Micron track sprayer. Analysis of the overall wall spray pattern using both fluorescein and IRS products demonstrated markedly less variation in the automated than in the manual spray. This confirms that a significant proportion of variation in spray application could be eliminated. Improved accuracy in insecticide application in trials would lead to more robust data and thus better-informed product development decisions, which could avoid unnecessary delays in bringing new products to the market.

With the exception of the study with BRF, the track sprayer resulted in a higher median concentration sprayed than manual spraying. For the insecticidal products, results were also compared to the target dose and showed underdosing with both spray methods. Although this underdosing was only significant for the track sprayer, it is likely that the underdosing was a factor for both methods, whilst the larger variation in manual spraying masked the difference between actual and target dose. Underdosing can occur if the speed of spraying is too fast, i.e., not enough liquid is deposited on the filter papers, or if the distance from the wall is too large. As both the distance and the speed are regulated for the track sprayer, it is unlikely that these factors caused the lower-than-expected application rate on the filter papers. It is recommended that in future studies, the track sprayer is used in conjunction with enhanced filter paper analysis so that any differences in the dose delivered compared to the target dose can be identified and evaluated.

To correct for potential deviations from target dose in the spray solution prior to spraying, we compared the sprayed filter papers to the concentration in liquid samples taken from the spray solution. Theoretically, the amount of insecticide sprayed onto filter papers would reflect the concentration of insecticide in the spray tank, assuming the spray nozzle moves at a constant speed up or down a swath and that the distance of the nozzle from the wall is also constant. Analysis of the spray solution can indicate dilution or mixing errors, such as adding too much or too little water or product to the spray tank or not shaking the spray tank to thoroughly mix the product with the water before and during spraying. We found that the concentration of PMM in the spray tank was considerably lower than expected, which may explain the lower-than-expected concentration found on filter papers with both spray methods.

Analysis of fluorescein values stratified by horizontal swath position showed that the 8002 nozzle used did not provide a consistent application rate across the horizontal swath, with less fluorescein being deposited in the centre of a swath. This difference was significant in the track sprayer deposits, but less apparent in the manual spray deposits. Similar to the results with fluorescein, the insecticide dose sprayed with the track sprayer was lower but more consistent in the centre position compared to positions at the edges of the swaths. Whilst the same nozzle was used for the track and manual spraying, different nozzles were used for fluorescein spray and each of the insecticidal products, making the possibility that this result could be an artefact of individual nozzles less likely.

We discerned a difference in spray rhythms in upward and downward swaths with manual spraying, even when the overall swath spray times were consistent between the two directions. The study was conducted using only one spray operator for each experiment and did not have the aim of characterising the entire range of variation that might be present during manual spraying. However, it is interesting to note that, even with expert training, and when keeping to the overall requirement of spraying 2 metres per 5 s, differences in rhythm can exist that lead directly to inconsistent spray application.

Height position analysis demonstrated significant differences in the amount of fluorescein applied between different wall height positions in the manual spray, particularly in the lower half of the wall. This aligns with the observation that spray operators tend to move the spray lance slowly at the top of the swath and then speed up towards the bottom. Although one significant difference was detected in fluorescein applied by the track spray between the uppermost and lowermost wall heights, no significant differences were seen when the overlap positions were also included, demonstrating that the track sprayer delivers a much more consistent spray application than the manual spray. Variation in the amount of insecticide sprayed manually was generally larger per vertical position compared to the track sprayer, but the trend of decreasing concentrations with lower wall heights was not shown when spraying with insecticides.

Overall, the track sprayer delivered a more consistent deposit of spray solution, making it a potential methodological improvement to experimental hut evaluations of novel IRS formulations.

## 5. Conclusions

Large variation was found in the amount of fluorescein and insecticide applied when following WHO guidelines for manual spraying by well-trained spray operators. When comparing the automated track sprayer to standard manual spraying, variation in application rates were significantly reduced in all instances, indicating that a large proportion of the variation in spray application can be eliminated by automating the spraying procedure.

## Figures and Tables

**Table 1:** HPLC methodology per active ingredient in the samples.

	Active Ingredient			
	Pirimiphos-Methyl	Broflanilide	Deltamethrin	Clothianidin
**Particle size**	5 µM	5 µM	5 µM	5 µM
**Wavelength**	232 nm	254 nm	232 nm	232 nm
**Run time**	22 min	22 min	9 min	9 min
**Mobile phase**	70% Acetonitrile: 30% water	70% Acetonitrile: 30% water	93% Acetonitrile: 7% water with 0.1% phosphoric acid	93% Acetonitrile: 7% water with 0.1% phosphoric acid
**Flow rate**	1 mL/min	1 mL/min	1 mL/min	1 mL/min

**Table 2:** Descriptive statistics for manual vs. track spraying using fluorescein. Results are given both with and without swath overlap positions.

Descriptive Statistic	Manual Spray Including Overlap	Manual Spray Not Including Overlap	Track Spray Including Overlap	Track Spray Not Including Overlap
**Minimum**	8.247	8.247	92.65	92.65
**Maximum**	663.4	416.3	302.8	267.4
**Median**	137.6	121.6	180.2	172.8
**Mean**	153.1	129.8	181.1	175.5
**SD**	88.83	69.51	36.15	33.87
**%CV**	58.03%	53.54%	19.96%	19.30%

**Table 4:** Horizontally stratified application rates of insecticides. Manual and track spray data was classified using left, centre, and right horizontal swath positions. Median application rate ± standard deviation is indicated for each horizontal position. Comparisons are done between horizontal positions by spray method. Significant differences are indicated by different lower-case letters.

		Left	Centre	Right
**BRF**	Manual	47.0 ^a^ ± 40.0	93.1 ^b^ ± 34.4	122.4 ^c^ ± 39.0
	Track	66.2 ^a^ ± 25.1	61.2 ^a^ ± 15.9	85.0 ^b^ ± 25.8
**PMM**	Manual	300.3 ^a^ ± 266.7	400.9 ^ab^ ± 233.9	446.0 ^b^ ± 292.1
	Track	782.6 ^a^ ± 228.5	462.5 ^b^ ± 67.2	467.9 ^b^ ± 138.5
**CTD**	Manual	205.7 ^a^ ± 134.4	151.7 ^b^ ± 80.4	168.8 ^ab^ ± 85.5
	Track	224.7 ^a^ ± 45.4	118.8 ^b^ ± 56.1	181.6 ^c^ ± 32.1
**DLT**	Manual	23.1 ^a^ ± 13.8	17.4 ^a^ ± 8.4	18.3 ^a^ ± 9.7
	Track	23.3 ^a^ ± 4.2	14.0 ^b^ ± 5.2	20.8 ^c^ ± 3.8

**Table 5:** Wall height analysis for track and manual spray for insecticides. Median application rate ± standard deviation is indicated for each vertical position. Comparisons are done between vertical positions by spray method. Significant differences are indicated by different lowercase letters.

		High	Mid-High	Centre	Mid-Low	Low
**BRF**	Manual	98.2 ^a^ ± 58.8	90.7 ^a^ ± 46.9	105.5 ^a^ ± 53.2	95.2 ^a^ ± 44.7	76.0 ^a^ ± 54.4
	Track	67.5 ^a^ ± 20.5	68.5 ^a^ ± 14.9	65.3 ^a^ ± 28.6	72.0 ^a^ ± 35.7	61.8 ^a^ ± 17.7
**PMM**	Manual	334.0 ^ab^ ± 169.4	538.3 ^b^ ± 317.8	215.1 ^a^ ± 172.5	421.1 ^ab^ ± 268.1	421.8 ^b^ ± 330.0
	Track	510.0 ^a^ ± 223.4	500.0 ^a^ ± 194.3	474.7 ^a^ ± 219.3	498.0 ^a^ ± 175.0	463.8 ^a^ ± 166.6
**CTD**	Manual	263.2 ^a^ ± 77.3	152.4 ^b^ ± 91.3	161.8 ^b^ ± 125.7	140.8 ^b^ ± 104.5	104.0 ^b^ ± 71.5
	Track	159.7 ^ab^ ± 53.9	148.8 ^a^ ± 36.6	194.9 ^ab^ ± 53.8	185.4 ^b^ ± 58.1	180.9 ^ab^ ± 46.5
**DLT**	Manual	31.6 ^a^ ± 8.8	16.8 ^b^ ± 9.9	19.2 ^b^ ± 12.8	16.4 ^b^ ± 9.5	11.7 ^b^ ± 7.4
	Track	17.0 ^ab^ ± 4.9	17.2 ^a^ ± 3.7	22.4 ^b^ ± 5.2	20.5 ^ab^ ± 5.9	19.6 ^ab^ ± 5.1

## Data Availability

The dataset generated and analysed during this study is available from the corresponding author upon reasonable request.
